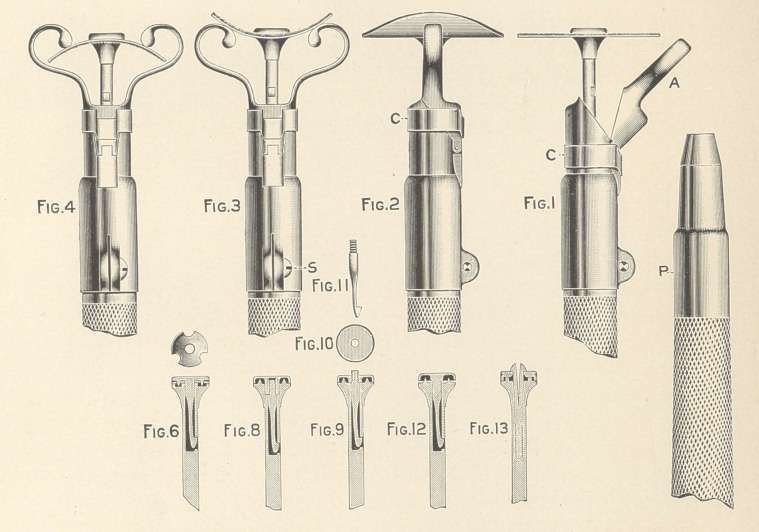# Dental Notes

**Published:** 1899-11

**Authors:** William Rollins

**Affiliations:** Boston, Mass.


### International Dental Journal.

VOL. XX. NOVEMBER, 1899. No. 11.

### Original Communications.[1]

### DENTAL NOTES.

BY WILLIAM ROLLINS, BOSTON, MASS.

NOTE III. THE A-W-L FLEXIBLE DISKS, DISK MANDRELS, AND DISK-BENDERS.

EARLY in my practice I saw the advantage of full contours in fillings. Therefore, soon after Green invented the dental engine, I turned my mind to finding some way to quickly finish the curved surfaces of fillings, inventing the flexible disk and disk-bender. Flexible disks are now made by the hundred thousand, and though they have been in use for a quarter of a century, it is still impossible to purchase good ones. The moment a disk gets wet the polishing powder comes off, because the glue is softened. In my first disks I overcame this defect by floating the paper from which the disks were to be cut on a solution of warm bichromated gelatin, drying, exposing to sunlight to harden the glue, washing to remove the excess of bichromate, and drying. It is to call attention to this method that I speak of flexible disks now. In the many years since disk-benders were devised, they have naturally undergone many changes, one now taking the place of two; serving to bend the disk away from the hand-piece, as shown in Figs. 2 and 3, or towards

[1] The editor and publishers are not responsible for the views of authors of papers published in this department, nor for any claim to novelty, or otherwise, that may be made by them. No papers will be received for this department that have appeared in any other journal published in the country.

it, as shown in Fig. 4. The disk-bender is shown with a hinge; open in Fig. 1, to insert a disk, closed in Figs. 2, 3, and 4. The ring (*C*) holds the swinging part in place. To have a disk-bender practical we need to be able to adjust its position on the hand-piece to give the disk more or less curvature. Hand-pieces are usually badly designed for the attachment of extra parts, because they taper. Every hand-piece should have a straight section, as shown in Fig. 1 (*P*), on which the disk-bender should fit, the screw (*S*) giving the necessary tension. The screw-disk mandrel, which until recently was the only practical one commercially available, was too slow in operation, so I invented a number of new ones. Where we do not exert a pull on the disk the beautiful mandrel, recently placed on the market by Dr. Maxwell, meets all requirements, but is not satisfactory with my disk-benders, as the disks pull free, so that I use my older forms, some of which are shown in Figs. 6 to 13. The one shown in Fig. 12 is the cheapest to make, as it has no adjustment for disks of different thickness. In Figs. 8 and 9 the adjustment for thickness is by means of a screw. In Fig. 8 the screw is out of sight, but this form is a little more expensive to make than the one represented in Figs. 9, 10, and 11. In Fig. 1 the releasing catch appears in side view. In Figs. 3 and 4 in front view. When it is desired to release a disk, the catch is pressed with the thumb-nail. The mandrel should be of tempered steel. It costs about a dollar and a half to make one of these mandrels, but if produced in quantity, the price need only slightly exceed that of the present inferior commercial forms. Notwithstanding the simplicity of these disk mandrels, I do not expect to see them in use until some manufacturer has patented them, so I have figured various forms, though I use but one, because I have observed that when a manufacturer patents a thing he did not invent he likes to make his patent as elaborate as possible.

## Figures and Tables

**Figure f1:**